# Genome-wide data implicate terminal fusion automixis in king cobra facultative parthenogenesis

**DOI:** 10.1038/s41598-021-86373-1

**Published:** 2021-03-31

**Authors:** Daren C. Card, Freek J. Vonk, Sterrin Smalbrugge, Nicholas R. Casewell, Wolfgang Wüster, Todd A. Castoe, Gordon W. Schuett, Warren Booth

**Affiliations:** 1 Department of Biology, The University of Texas Arlington, Arlington, TX USA; 2 Department of Organismic and Evolutionary Biology, Harvard University, Cambridge, MA USA; 3 Museum of Comparative Zoology, Harvard University, Cambridge, MA USA; 4 Naturalis Biodiversity Center, Leiden, The Netherlands; 5 Amsterdam Institute of Molecular and Life Sciences, Division of BioAnalytical Chemistry, Department of Chemistry and Pharmaceutical Sciences, Faculty of Science, Vrije Universiteit Amsterdam, 1081HV Amsterdam, The Netherlands; 6 Wildlife Ecology and Conservation Groups, Wageningen University, Wageningen, The Netherlands; 7 Centre for Snakebite Research and Interventions, Liverpool School of Tropical Medicine, Liverpool, UK; 8 Molecular Ecology and Evolution Group, School of Biological Sciences, Bangor University, Bangor, UK; 9 Chiricahua Desert Museum, Rodeo, NM USA; 10 Department of Biology, Neuroscience Institute, Georgia State University, Atlanta, GA USA; 11 Department of Biological Science, The University of Tulsa, Tulsa, OK USA

**Keywords:** Developmental biology, Evolution, Genetics

## Abstract

Facultative parthenogenesis (FP) is widespread in the animal kingdom. In vertebrates it was first described in poultry nearly 70 years ago, and since then reports involving other taxa have increased considerably. In the last two decades, numerous reports of FP have emerged in elasmobranch fishes and squamate reptiles (lizards and snakes), including documentation in wild populations of both clades. When considered in concert with recent evidence of reproductive competence, the accumulating data suggest that the significance of FP in vertebrate evolution has been largely underestimated. Several fundamental questions regarding developmental mechanisms, nonetheless, remain unanswered. Specifically, what is the type of automixis that underlies the production of progeny and how does this impact the genomic diversity of the resulting parthenogens? Here, we addressed these questions through the application of next-generation sequencing to investigate a suspected case of parthenogenesis in a king cobra (*Ophiophagus hannah*). Our results provide the first evidence of FP in this species, and provide novel evidence that rejects gametic duplication and supports terminal fusion as a mechanism underlying parthenogenesis in snakes. Moreover, we precisely estimated heterozygosity in parthenogenetic offspring and found appreciable retained genetic diversity that suggests that FP in vertebrates has underappreciated evolutionary significance.

## Introduction

Asexual reproduction in otherwise sexually reproducing species—a phenomenon termed facultative parthenogenesis (FP)^1^—has been reported in three major lineages of vertebrates: chondrichthyan fishes^2–4^, birds^5,6^, and squamate reptiles^7,8^. Originally limited to a few instances in captivity, FP has been described as an outcome of reproductive error^1,9^. However, reports of vertebrate FP continue to increase both in number and taxonomic diversity, including cases from the wild^3,10^. With this accumulating information, our understanding of this reproductive mode has expanded greatly^8^, and commonalities have been detected across all vertebrate lineages shown capable of FP. Important commonalities include: (i) constraints that the sex chromosome system itself places on the sex of parthenogens^8,11^, (ii) the capacity to switch between sexual and asexual modes^8,12^, and (iii) the ability to produce consecutive (i.e., multiple) FP births^5,13–18^. Recently, second generation FP (i.e., parthenogenetic reproduction by a parthenogen) has been documented in the white-spotted bamboo shark, *Chiloscyllium plagiosum*^19^, and observed in the royal python, *Python regius* [W. Booth, unpubl. data]. Furthermore, a parthenogenetic boa constrictor, *Boa imperator*, was found to be capable of sexual reproduction [W. Booth. unpubl. data]. Combined, these diverse data strengthen the view that FP is not an uncommon mode of reproduction in certain vertebrate taxa, and suggest that its significance in vertebrate evolution has been underestimated^8^.

**Figure 1:**
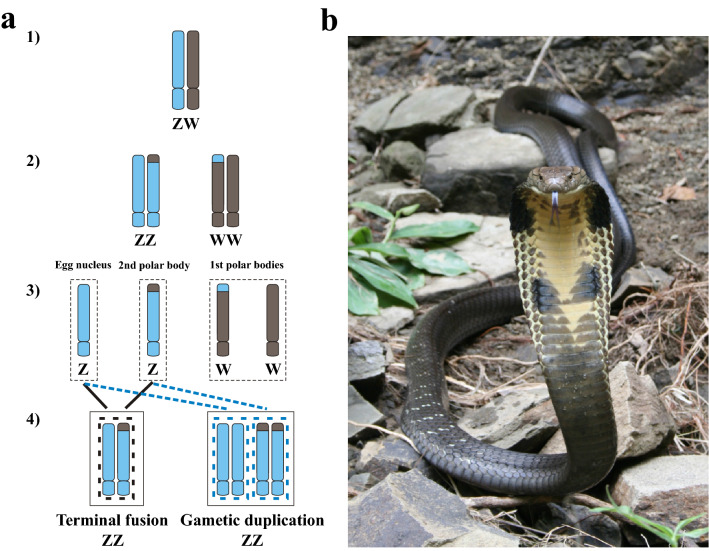
(**a**) Proposed mechanisms of automixis in snakes. (1) Primordial germ cell. (2) Meiotic products following DNA replication and recombination during the first round of cell division. (3) Meiotic products following the second round of cell division. (4) Potential sex chromosomal arrangements following terminal fusion and gametic duplication (here depicted for one Z chromosome). Note that parthenogens with a WW sex chromosome arrangement are not, at present, known to be viable. (Modified from^58^). (**b**) Adult king cobra (*Ophiophagus hannah*). Photo courtesy of Freek Vonk.

The developmental mechanism responsible for nearly all cases of vertebrate FP has been ascribed to automixis, with restoration of diploidy through the fusion or duplication of meiotic products. Of the automictic modes, terminal fusion (TF) is the most commonly cited mechanism^1,8^. Under this mode, the egg nucleus fuses with its second polar body, resulting in the retention of reduced levels of heterozygosity limited to the telomeric regions of chromosomes^1,8^ (Fig. 1a). Few studies, however, provide supporting evidence, i.e., heterozygosity in FP progeny^17,18,20^. Gametic duplication (GD), in contrast, is predicted to result in genome-wide homozygosity^1,8^ (Fig. 1a). Accurately assigning which mechanism underlies FP in a given species has proven challenging, largely owing to limitations of the molecular markers employed (mainly microsatellites). In such studies, a dramatic reduction in heterozygosity has been shown in parthenogens, relative to their mother, with most parthenogens exhibiting homozygosity across all loci surveyed^2,3,7,12–17,21–25^. Consequently, due to levels of heterozygosity in the parthenogens, some authors infer GD^25^, whereas others propose TF^2,8,17,18^. A key issue with the conclusions of these studies is that there is limited power to detect low levels of retained heterozygosity expected under TF with only a few markers employed. Thus, high false positive rates may result for identifying GD over TF. Unfortunately, most published studies on the phenomenon of FP have not been sufficiently robust to accurately differentiate between these two developmental mechanisms and provide little opportunity to examine secondary questions focused on how genetic variation is inherited in parthenogenetic offspring, and the impact parthenogenesis may have on the evolutionary trajectories of natural populations^8,25^.

Arguably, with such indeterminate outcomes, understanding which mechanism of automixis is operating has important implications on the levels of genetic diversity retained in parthenogenetic offspring, which in turn has major ramifications for understanding the impact that these parthenogens might have on the genetic diversity of natural populations. This consideration is particularly significant when the status of a species, or population, is threatened or endangered^3,7^. These concerns are further amplified by the findings that FP may be heritable^5,6^, which could lead to greater incidence of this phenomenon in small populations as a result of inbreeding^3^. Despite advances that recent studies have made towards understanding FP in vertebrates^9^, the fundamental questions of: (i) resolving which automictic mechanism(s) underlie FP, and (ii) how FP impacts individual-level genetic diversity on a genome-scale, remain unresolved.

Compared to other vertebrate lineages, recent studies in snakes have generated novel and field-advancing results that have significantly contributed to our understanding of FP^8,11,26^; thus, snakes represent an ideal model system with which to address outstanding questions. Following the publication of its genome^27^, the king cobra, *Ophiophagus hannah* (Fig. 1b), has emerged as a new model system for studying multiple aspects of snake evolution, development, and venom. This iconic species, distributed widely across South and Southeast Asia, is the world’s longest venomous snake, attaining lengths up to 5 m. Owing to human persecution and habitat destruction, it is of high conservation concern throughout its natural range, and IUCN-listed as 'Vulnerable'^28^. In an effort to address the outstanding questions in vertebrate FP, we used the king cobra as a model to explore genetic patterns of tens of thousands of loci from a virgin king cobra female and her two male offspring suspected of being parthenogens. Restriction Associated DNA sequencing (RADseq) data provide an unprecedented, genome-scale view of the impact of FP on genetic diversity and inheritance, confidently providing support for a terminal fusion-based mechanism of development that results in the maintenance of appreciable levels of heterozygosity in parthenogenetic offspring.

## Materials and methods

### Subject history and sampling

The mother (Cobra UK) was an adult, originally imported to Germany from Sumatra as a 70 cm juvenile in September 2012. Kept in isolation, on 3 August 2015, at ~ 200 cm, she laid 24 eggs. Two eggs were viable, determined through the detection of blood vessels following egg candling, and were artificially incubated in a Jaeger incubator at 27–28 °C in a substrate consisting of moist vermiculite. One egg was opened on 26 October 2015, revealing a male offspring (offspring #1—Cobra211), which died two days later. The second egg, opened on 12 November 2015, also contained a male offspring (offspring #2—CobraFV807), which died within three days. Both fetuses were sexed based on morphology and were undersized (offspring 1—27.7 cm; offspring 2–20.7 cm, Supplementary Figure 1a) compared to an average expected hatchling size of ~ 50 to 55 cm^29^. In cases of facultative parthenogenesis (FP) involving several lineages of colubroid snakes, particularly natricines (e.g., genera *Nerodia* and *Thamnophis*) developmental abnormalities to both the head region and or body are not uncommon^8,17,21,30^. These deformities include, but are not limited to, incomplete development of the brain, impartial fusion of the cranium, uni- or bilateral absence of one of both eyes (anophthalmia), loss or the addition of body scales, scoliosis of the vertebral column. The craniofacial abnormalities observed in the neonate king cobras are similar to those described for the checkered garter snake (Supplementary Figure 1b^8^). Allen et al*.*^20^ reported similar post-cranial developmental abnormalities (e.g., vertebral scoliosis) associated with FP in an elapid species, the death adder (*Acanthophis antarcticus*). DNA was extracted from shed skin from the mother and muscle tissue from the offspring using standard phenol–chloroform methods. All extractions were quantified using Qubit broad-range DNA assays (Thermo Fisher Scientific) following the manufacturer’s instructions. *Ophiophagus hannah* is a widespread species with high morphological and genetic variability^31,32^, and likely constitutes a species complex. A taxonomic revision is in progress (P. Gowri Shankar et al., in prep). To future-proof the identifiability of our specimen in relation to future taxonomic changes, we Sanger-sequenced parts of the mitochondrial genes for NADH dehydrogenase subunit 4 (ND4) and 16S ribosomal RNA of the mother (as used by Gowri Shankar et al.) as barcode vouchers. For primers and PCR conditions see Maddock et al.^33^.

### Genomic library generation and data processing

A modified version of the Peterson et al.^34^ protocol was used to prepare double digest RADseq libraries. Genomic DNA was simultaneously cut with both rare *Pst*I (6 bp) and common *Sau*3AI (4 bp) restriction enzymes, estimated to target approximately 200,000 genomic loci per individual. To allow for hierarchical pooling and multiplexing of samples, barcoded Illumina adapter oligonucleotides were ligated to the ends of digested DNA. These adapters also included 8 bp unique molecular identifiers (UMIs; 8 consecutive random nucleotides prior to the ligation site). Following adapter ligation, samples were pooled, and these pools were size selected for a range of 430–600 bp using a Blue Pippin (Sage Science). After size selection, samples were PCR-amplified with pool-specific indexed primers, and amplification products were further pooled into a single sample based on molarity calculations from analysis on a Bioanalyzer (Agilent Technologies) using a DNA 7500 chip. The final pooled libraries were sequenced on an Illumina HiSeq 2000 lane, resulting in at least 2 million reads per sample (Table 1).

**Table 1:** Measures of the number of paired-end reads mapped to the king cobra, *O. hannah*, reference genome, the number of SNPs vs. the reference genome in final thinned genotype dataset, mean heterozygosity (*OH *observed heterozygosity, *HL *homozygosity by loci), and mean pairwise relatedness for all samples. Values above the diagonal in the relatedness matrix represent *B*_xy_, while values below the diagonal represent *M*_xy_. *SD *standard deviation.

Sample	Mapped PE reads	SNPs	Mean (± SD) heterozygosity		Mean (± SD) pairwise relatedness		
			OH	HL	Mother	Offspring #1	Offspring #2
Mother (Cobra UK)	28,844,748	401	0.011 (± 0.004)	0.30 (± 0.027)	–	0.99 (± 0.0004)	0.99 (± 0.0004)
Offspring #1 (Cobra211)	2,696,748	373	0.007 (± 0.005)	0.53 (± 0.027)	0.99 (± 0.0004)	–	0.99 (± 0.0005)
Offspring #2 (CobraFV807)	24,027,342	328	0.007 (± 0.001)	0.52 (± 0.029)	0.99 (± 0.0004)	0.99 (± 0.0005)	–

### Read processing and genotyping

Raw Illumina sequence data were filtered to remove PCR clones using the adapter UMIs and the clone_filter tool from the Stacks v. 1.35 analysis pipeline^35,36^. Samples were parsed using the process_radtags tool from Stacks, with restriction sites and barcodes rescued using default settings, and Trimmomatic v. 0.32^37^ was used to quality filter the resulting data using the settings LEADING:10 TRAILING:10 SLIDINGWINDOW:4:15 MINLEN:36. An iterative strategy was used to focus on genetic variation sampled from high-quality RAD loci present in all individuals. Quality-trimmed reads were mapped to the *Ophiophagus hannah* genome (NCBI version OphHan1.0^27^) using the mem algorithm in BWA v. 0.7.12-r1039^38^. SAMtools v. 1.3.1^39,40^ and Picard v. 1.106 were used to process mapping files for each sample and merge mappings for downstream analyses. GATK v. 3.8-0-ge9d806836^41–43^ was used to perform realignment around indels with default settings. The mapping data was then used to isolate the coordinates of RAD loci present in all individuals by identifying high quality (Phred ≥ 30) sites where read depth was at least 5 per individual, merging adjacent sites together to form loci (allowing up to 10 bp low coverage “gaps” in coordinates), and filtering away loci shorter than 100 bp. Using these RAD loci footprint coordinates, a new reference sequence from the *O*. *hannah* reference genome was exported using BEDtools v. 2.29.0^44^ that only contained the RAD locus regions.

For genotyping, a second round of mapping was conducted and variant calling performed using the GATK best-practices guidelines^41–43^. BWA was used to map quality-trimmed reads to the RAD locus reference and SAMtools, Picard, and GATK were used to quality control and realign around indels, as above. Variants were called using GATK HaplotypeCaller using default settings by first producing GVCF variant files for individuals before performing joint genotyping across all samples. Variants were filtered using BCFtools and VCFtools v. 0.1.15^45^ as follows: (1) indels were excluded; (2) genotype calls for individuals with a read depth of less than 5 were set as missing data; (3) SNPs within 3 bp of indels and clusters of indels separated by 10 bp or less were excluded; (4) variants with a Phred quality score below 30 were excluded; (5) SNPs with significant statistical biases were removed using the hard filter ‘QD < 2 || FS > 60.0 || MQ < 40.0 || MQRankSum < − 12.5 || ReadPosRankSum < − 8’; (6) SNPs with a total depth greater than 2 × average depth (= 100) and less than 0.5 × average depth (= 25) were excluded; and (7) non-biallelic SNPs were excluded. We further thinned SNPs to avoid the potential effects of linkage by randomly selecting one variant per 50 kb region of the king cobra genome.

### Genomic analyses of patterns of parthenogenesis

Filtering of loci (by quality, representation across individuals, and by linkage) resulted in a dataset of 20,562 independent (i.e., unlinked) RAD loci with genotypes called in the mother and both offspring. Given a genome assembly size of approximately 1.6 Gb, we expect a RAD locus density of one marker per 80 kb. The Rhh^46^ package in R v. 3.4.3^47^ was used to calculate two measures of genome-wide heterozygosity for each individual: proportion observed heterozygosity (OH; 0 is complete homozygosity and 1 is complete heterozygosity) and homozygosity by loci (HL^48^; 0 is complete heterozygosity and 1 is complete homozygosity). Two measures of pairwise relatedness were calculated between all samples using the R package Demerelate^49^: the shared alleles index (*B*_xy_^50^) and genotype sharing (*M*_xy_^51^)—both measures vary between 0 (no relatedness) and 1 (complete relatedness or identical genotypes). A custom R function available from https://github.com/darencard/parthenogenesis was used to sample 100 bootstrap datasets to measure variance in each measure. The proportions of retained and lost maternal heterozygosity in each offspring was quantified and we used a binomial test to determine whether there were equal proportions of loci that are homozygous for the reference (0/0) or alternative (1/1) allele at loci where heterozygosity was lost in offspring, as is expected under a model of independent inheritance of unlinked loci. We estimated the Jaccard index to quantify patterns of retained heterozygosity shared in both offspring in the empirical dataset compared to randomly permutated datasets based on the formula $J(A,\;B) = \frac{{\left| {A \cap B} \right|}}{| A \cup B|}$ where *A*, and *B* represent sets of loci in the two offspring.

## Results and discussion

**Figure 2:**
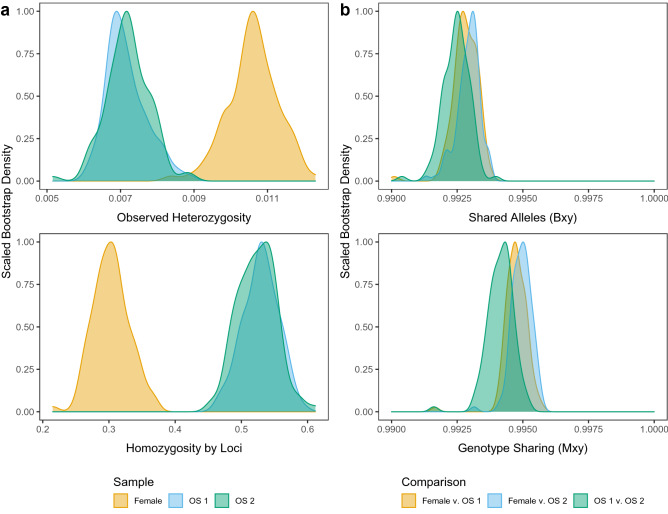
(**a**) Bootstrap densities of two measures of individual heterozygosity for each member of the cobra family (female = mother, OS 1 = offspring 1, OS 2 = offspring 2) based on 100 bootstrap replicates of 20,562 independent RAD loci: (top) Proportion of heterozygous sites (Observed Heterozygosity), and (bottom) the standardized level of homozygosity (Homozygosity by Loci). (**b**) Bootstrap densities of two measures of pairwise relatedness between the mother and each parthenogenetic offspring based on 100 bootstrap replicates sampled variants: (top) shared alleles index (*B*_xy_) and (bottom) genotype sharing index (*M*_xy_). Sexual reproduction would produce measures at 0.5.

Here we provide the first in-depth, genome-wide assessment of genetic diversity for a squamate reptile reproducing via FP, through the comparison of two parthenogenetic male king cobra siblings with their mother. The analysis of thousands of loci from throughout the genome indicates that both offspring had significantly reduced levels of heterozygosity relative to the mother, though offspring #2 retained slightly higher amounts than offspring #1 (Fig. 2a, Table 1). Both measures of relatedness fell between approximately 0.99 and 1 (Fig. 2b, Table 1), which is far above the expectation of 0.5 in pairwise comparisons between a parent and sexually produced progeny. The highly reduced level of heterozygosity, inflated values of pairwise relatedness, and offspring sex (i.e., male being the expected sex of a parthenogen in species with ZZ/ZW sex determination; see^8,11^), strongly support the conclusion that these offspring are the product of FP, and provide the first evidence of this mode of reproduction in the king cobra.

### Automictic mechanisms and evolutionary consequences of FP

Resolving the automictic mechanism underlying the development of progeny via FP in previous studies has relied on the level of homozygosity observed in the progeny. For example, terminal fusion automixis is expected to produce offspring that exhibit highly elevated levels of homozygosity relative to the mother, with the majority of heterozygous loci in the mother becoming differentially homozygous in the offspring. In contrast, central fusion automixis is predicted to produce parthenogens that retain levels of heterozygosity comparable to the mother. However, resolving the mechanism between terminal fusion and gametic duplication has proven difficult, for the latter results from the duplication of the gametes chromosomes and hence 100% homozygosity (Fig. 1a). As terminal fusion is assumed to result in low levels of heterozygosity retained at the telomeric regions, detection of heterozygosity relies on having markers in these specific regions of recombination. Previous studies of vertebrate FP have largely utilized microsatellite markers for genetic confirmation. However, the limited numbers of markers employed in each study and the low likelihood that these markers will be located in regions of recombination has resulted in parthenogens exhibiting complete homozygosity at the mother’s heterozygous loci in all but two studies^17,18^. Accordingly, while TF has been cited as the most likely developmental mechanism^8^, GD also has been proposed^25^.

Using RADseq, we conclusively show, through the presence of retained heterozygosity in FP offspring, that the developmental mechanism of automixis is TF over GD. Here, the detection of low, but clearly identifiable levels of heterozygosity in each offspring (Fig. 2a), is in agreement with TF. Notably, approximately a quarter of maternal heterozygosity is retained in these offspring (N = 278 maternal heterozygous loci; 23% retained in offspring #1 and 27% retained in offspring #2), which is greater than previous estimates of retained heterozygosity based on microsatellite markers (typically 0% retained heterozygosity^21,22,25,52^). This discrepancy may stem from technical differences between SNP and microsatellite markers but given that non-trivial retained heterozygosity is also observed in a far more stringently filtered set of SNPs (Phred quality score > 1000; N = 86 maternal heterozygous loci; 20–22% retained heterozygosity), we speculate that previous studies based on small numbers of microsatellite loci had reduced power to detect retained heterozygosity. Further exploration of FP using genomic datasets is necessary to better understand how much maternal heterozygosity is retained in parthenogenetic offspring. Moreover, affirming a pattern of significant retention of heterozygosity will require reconciling our current understanding of the impact of FP on genetic diversity and the evolutionary trajectories of natural populations.

### Patterns of inheritance of genome-wide heterozygosity

**Figure 3:**
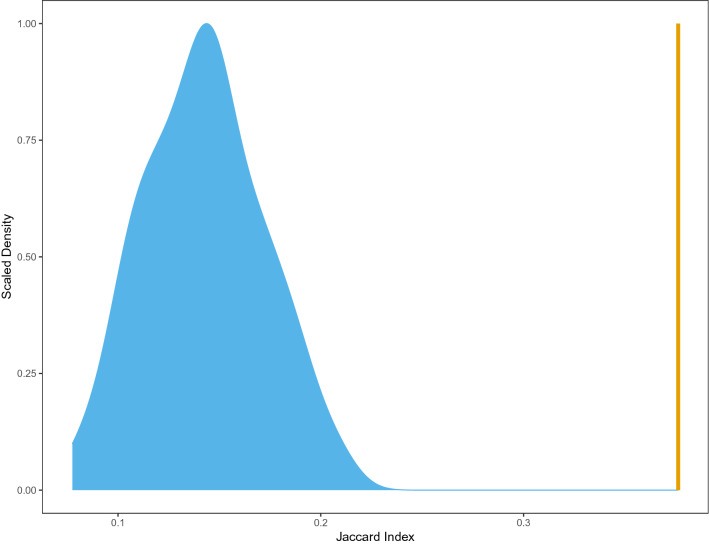
Scaled density of Jaccard index measures of the overlap in heterozygous sites in the two offspring, showing that the degree of overlap in the empirical dataset (in gold) is nonrandom and significantly greater than expected based on measures from 100 randomly permutated datasets (in blue).

Genome-wide patterns of inheritance of heterozygosity have never been quantitatively compared across multiple offspring due to low numbers of profiled markers or a lack of sibling samples necessary for evaluating inheritance. Under a model of terminal fusion automixis, we expected that the modest amount of heterozygosity retained only in the telomeric regions of chromosomes would result in modest amounts of shared retained heterozygosity between the two offspring. In accordance with our expectations, while each of the offspring in this study does possess unique genomic regions with retained heterozygosity, a large percentage of retained heterozygosity is shared between offspring (38%), which is greater than expected by chance (Fig. 3). The degree of shared heterozygosity between offspring confidently rules out false positive signals of retained heterozygosity due to genotyping error and is likely explained by localization of heterozygosity to similar genomic regions—likely the distal ends of chromosome arms, which are predicted to represent regions of retained heterozygosity in TF. Unfortunately, it is not possible to directly localize regions of retained offspring heterozygosity and confirm their location in the telomeric regions of chromosomes due to the low contiguity of the current king cobra genome assembly^27^. However, more indirect measures of genomic composition may provide information on the locations of regions of retained heterozygosity, providing further support for a model of terminal fusion (TF) automixis in this species. Under the TF automixis model, GC content is expected to be inflated around areas of retained heterozygosity due to higher rates of recombination and GC-biased gene conversion in telomeric regions (Fig. 1a)^53–55^. However, we find that GC content in regions surrounding retained heterozygosity in both offspring is statistically indistinguishable from GC content in regions that lose heterozygosity in the offspring and from permutated datasets produced from regions homozygous in the mother and both offspring (Supplementary Materials).

Terminal fusion automixis does result in the retention of maternal heterozygosity in offspring, but the majority of heterozygosity found in centromeric regions ends up purged in the genomes of offspring (Fig. 1a). Under any form of automixis, roughly equal numbers of loci that are heterozygous in the mother will segregate as homozygous genotypes of one of the parental alleles in the offspring^1,8^. Yet, in our dataset, we observed biased patterns of inheritance in both of the offspring, where the reference allele is preferentially inherited (Table 2, Supplementary Figure 2). While these results are not immediately predicted by any existing model of automixis, this observation may be explained by some form of biased gene conversion^53,56,57^, a process which has not been thoroughly investigated in snakes. Recent studies on the evolution of mammalian genomic landscapes supports such non-Mendelian transmission of alleles to occur in regions located near recombination sites that yield a GC bias, and thus result in an evolutionary advantage for GC over AT alleles^57^. While we did not observe a bias in GC content in regions of retained heterozygosity in the king cobra genome (Supplementary Materials, Supplementary Figure 3), this may be due to the limitations of the dataset and these findings do not refute TF as the developmental mechanism underlying facultative parthenogenesis in snakes^8^. Other unknown mechanisms or technical artifacts may therefore explain the biased pattern of inheritance in the two offspring. Indeed, the large bias towards inheritance of the reference allele (i.e., the allele present in the reference genome) in both offspring is not expected under GC-biased gene conversion and is logical in a situation where there is reduced power to call alternative SNPs (alleles) due to low sequencing coverage or sample numbers. This pattern may therefore be a result of a statistical artifact, but additional studies that leverage high-coverage, genome-wide data in larger numbers of samples should provide a better opportunity to further explore the biased patterns of inheritance observed in this study.

**Table 2:** A tally of the fate of maternal heterozygous loci (N = 278) in both offspring. 0/0, 0/1, and 1/1 encode homozygous and heterozygous locus genotypes based on the reference genome (0) and alternative alleles (1). The Binomial Test *p*-value column represents the results of two-sided binomial tests for each offspring that compared the proportions of 0/0 vs. 1/1 genotypes under the assumption of an expected proportion of 0.5 for each with random inheritance of unlinked loci.

Offspring	No. of 0/0 loci	No. of 0/1 loci	No. of 1/1 loci	Binomial test *p*-value
Offspring #1	150	64	64	3.9 × 10^–9^
Offspring #2	144	75	59	2.2 × 10^–9^

## Conclusion

In summary, here we used a genome-scale dataset to study parthenogenesis in a non-traditional model vertebrate and find conclusive support for terminal fusion automixis-mediated facultative parthenogenesis in the king cobra. When considered alongside genomic data recently reported in two species of Australian elapid^20^, and microsatellite data presented in a North American garter snake^17^, this likely represents the underlying developmental mechanism in all species of snakes presently reported to have reproduced through facultative parthenogenesis^8^. Our genomic dataset also illuminates patterns of inheritance during TF, including the first precise estimates of appreciable retained heterozygosity in parthenogenetic offspring that have important biological and evolutionary implications. These results advance our understanding of FP in vertebrates and identify new areas of research for which snakes, and likely other squamate reptiles, represent a robust model system.

### Ethics

Specimens were maintained in a private collection (see Acknowledgments), with tissues collected non-invasively (female shed skin) or post-mortem (offspring).

## Supplementary Information

Supplementary Figure 1.

Supplementary Figure 2.

Supplementary Figure 3.

Supplementary Information.

## Data Availability

Raw Illumina data are available from the NCBI SRA (accessions SRR13866658–SRR13866660). Genotype data (including VCF) and measures of heterozygosity, relatedness, and GC content are available from Figshare (https://doi.org/10.6084/m9.figshare.14166677) and R analysis functions for calculating heterozygosity and relatedness are available from https://github.com/darencard/parthenogenesis. Mitochondrial 16S and ND4 sequences are available from GenBank (accession MT946535-6).
