# Supplementary figures and images for: Genome-wide data implicate terminal fusion automixis in king cobra facultative parthenogenesis
Source: Sci Rep. 2021 Mar 31;11:7271. doi: 10.1038/s41598-021-86373-1 (PMC8012631; doi:10.1038/s41598-021-86373-1)

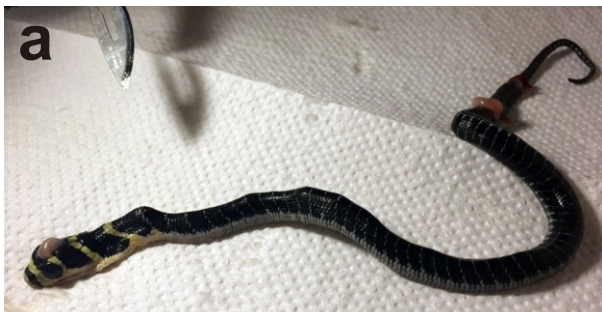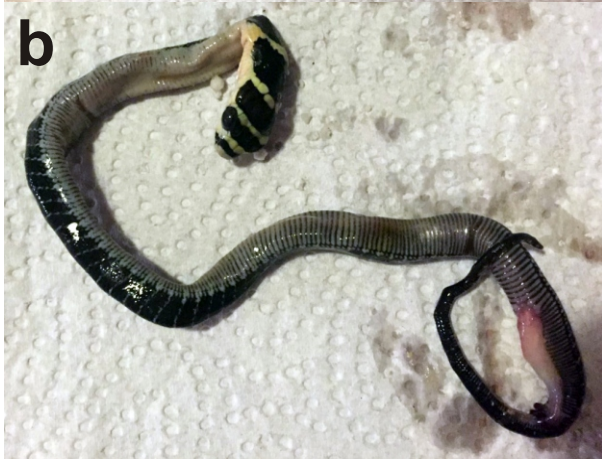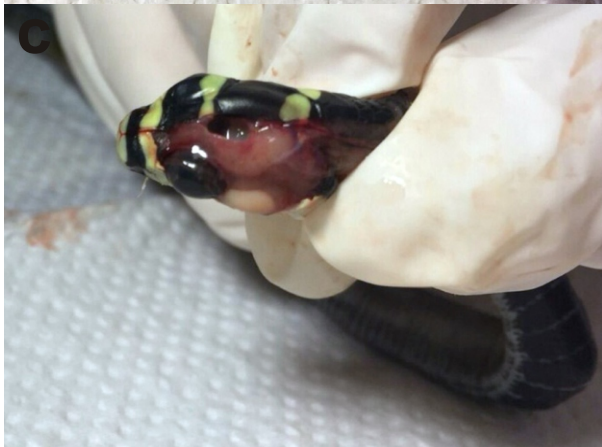

Supplement: Supplementary file 1 — Supplementary Figure 1. [file 41598_2021_86373_MOESM1_ESM.pdf]

Loci Proportions

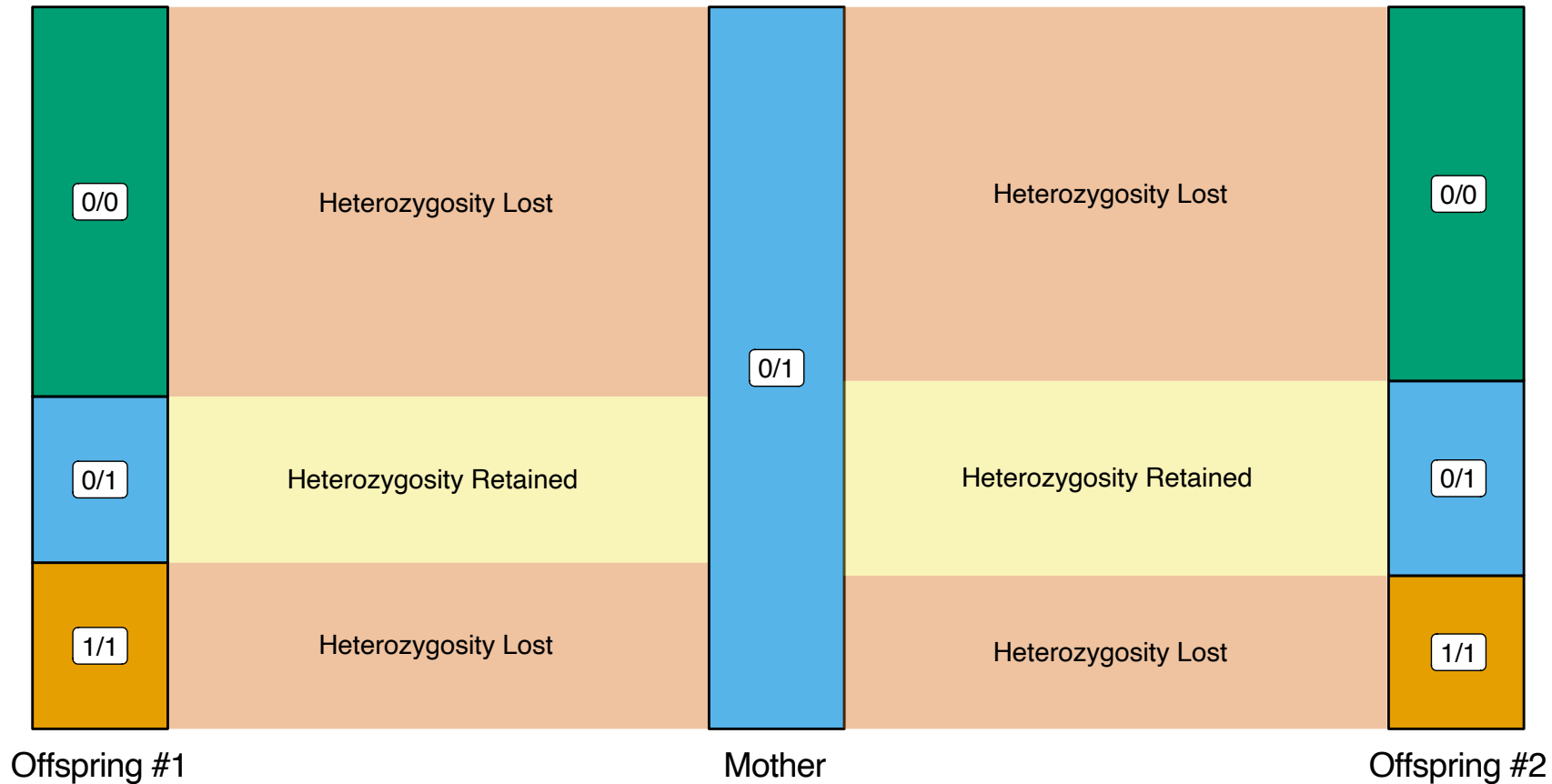

Supplement: Supplementary file 2 — Supplementary Figure 2. [file 41598_2021_86373_MOESM2_ESM.pdf]
