# Supplementary material for: Genome-wide data implicate terminal fusion automixis in king cobra facultative parthenogenesis
Source: Sci Rep. 2021 Mar 31;11:7271. doi: 10.1038/s41598-021-86373-1 (PMC8012631; doi:10.1038/s41598-021-86373-1)
Supplement: Supplementary file 3 — Supplementary Figure 3. [file 41598_2021_86373_MOESM3_ESM.pdf]

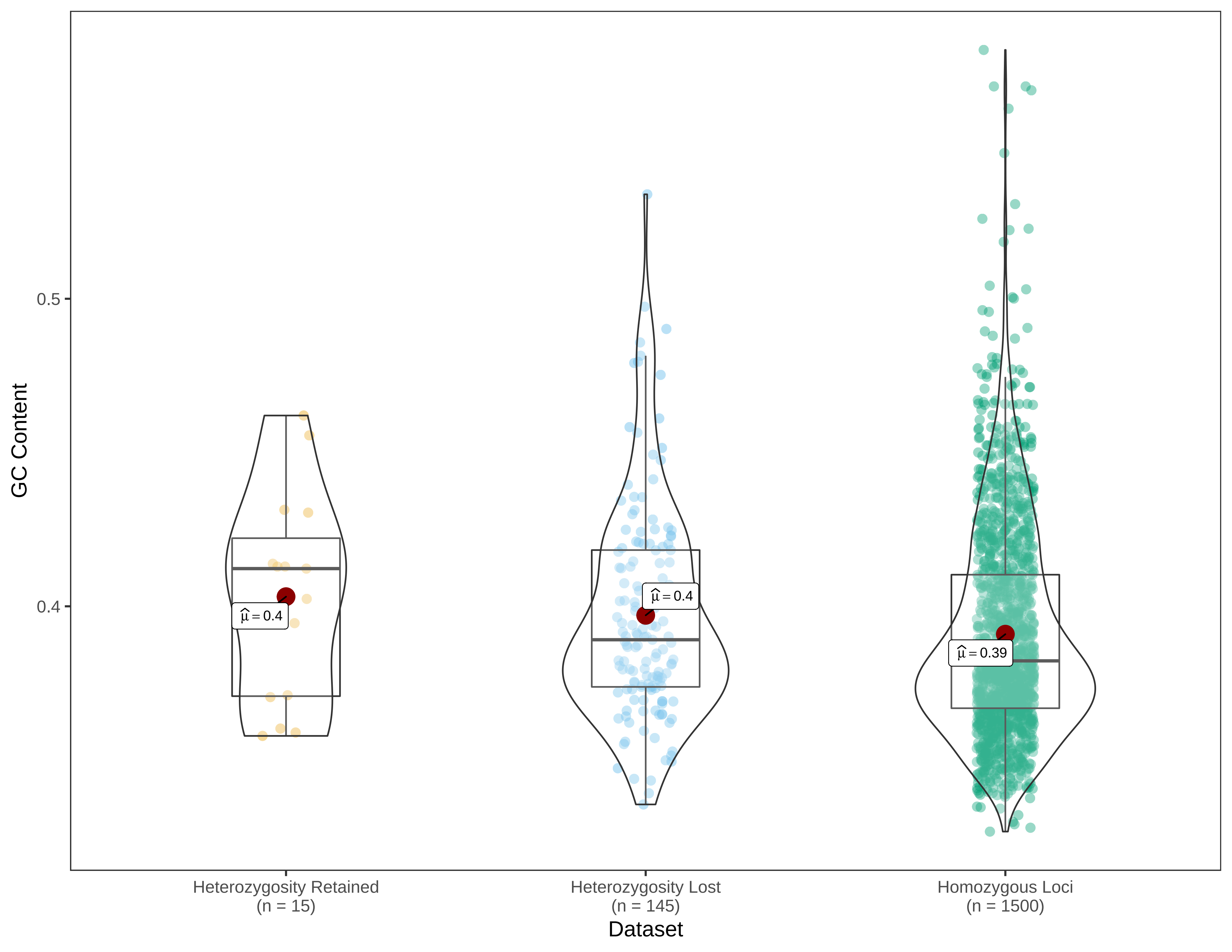

GC Content
0.5
0.4
μ̂ = 0.4
μ̂ = 0.4
μ̂ = 0.39
Heterozygosity Retained
(n = 15)
Heterozygosity Lost
(n = 145)
Homozygous Loci
(n = 1500)
Dataset
