# Supplementary material for: Genome-wide data implicate terminal fusion automixis in king cobra facultative parthenogenesis
Source: Sci Rep. 2021 Mar 31;11:7271. doi: 10.1038/s41598-021-86373-1 (PMC8012631; doi:10.1038/s41598-021-86373-1)
Supplement: Supplementary file 4 — Supplementary Information. [file 41598_2021_86373_MOESM4_ESM.docx]

***Supplementary Materials***

**Genome-wide data implicate terminal fusion automixis in king cobra facultative parthenogenesis**

Daren C. Card^1,2,3^, Freek J. Vonk^4,5^, Sterrin Smalbrugge^6^, Nicholas R. Casewell^7^, Wolfgang Wüster^8,9^, Todd A. Castoe^1^, Gordon W. Schuett^9,10^, and Warren Booth^8,11,*^

^1^ Department of Biology, The University of Texas Arlington, Arlington, Texas, USA.

^2^ Department of Organismic and Evolutionary Biology, Harvard University, Cambridge, MA, USA.

^3^ Museum of Comparative Zoology, Harvard University, Cambridge, MA, USA.

^4^ Naturalis Biodiversity Center, Leiden, The Netherlands.

^5^ Amsterdam Institute of Molecular and Life Sciences, Division of BioAnalytical Chemistry, Department of Chemistry and Pharmaceutical Sciences, Faculty of Science, Vrije Universiteit Amsterdam, Amsterdam 1081HV, The Netherlands

^6^ Wildlife Ecology and Conservation Groups, Wageningen University, Wageningen, The Netherlands

^7^ Centre for Snakebite Research & Interventions, Liverpool School of Tropical Medicine, Liverpool, UK.

^8^ Molecular Ecology and Evolution Group, School of Biological Sciences, Bangor University, Bangor, UK.

^9^ Chiricahua Desert Museum, Rodeo, New Mexico, USA.

^10^ Department of Biology, Neuroscience Institute, Georgia State University, Atlanta, Georgia, USA.

^11^ Department of Biological Science, The University of Tulsa, Tulsa, Oklahoma, USA.

^*^ **Corresponding author**: Warren Booth – warren-booth@utulsa.edu

## Supplementary Materials & Methods and Results

The low contiguity of the current king cobra genome assembly prevents the direct localization of regions of retained offspring heterozygosity, restricting our ability confirm the location of retained heterozygosity in the telomeric regions of chromosomes. However, more indirect measures of genomic composition may provide information on the locations of regions of retained heterozygosity, providing further support for a model of terminal fusion (TF) automixis in this species. Under the TF automixis model, GC content is expected to be inflated around areas of retained heterozygosity. This pattern stems from the increased rates of recombination in the terminal ends of chromosome arms, which tend to be higher in GC content overall ^[1–5]^. Indeed, GC content is known to be elevated near the ends of chromosomes in snake genomes ^[6–8]^. Therefore, local patterns of GC content were assessed in 50 kb windows surrounding RAD loci with retained heterozygosity in both offspring, RAD loci where heterozygosity is lost in the offspring, and randomly permutated regions homozygous in the mother and both offspring. In all cases, to reduce bias from loci located on short assembly scaffolds or in regions with large gaps, estimates of GC content were only measured if the number of non-N bases within the genomic window was at least 20,000. We used a Kruskal–Wallis one-way ANOVA ^[9]^ to compare the empirical and permutated distributions, with *post hoc* pairwise comparisons made using a Dwass-Steel-Crichtlow-Fligner test ^[10]^ with a Benjamini-Hochberg correction for multiple tests ^[11]^. We calculated the rank Epsilon squared measure to quantify effect size ^[12]^.

In accordance with our expectations, we find that GC content in regions surrounding retained heterozygosity in both offspring is statistically distinguishable from GC content in regions that lose heterozygosity in the offspring, and distinct from permutated datasets produced from regions homozygous in the mother and both offspring (χ^2^ [2] = 7.51, *p* = 0.023), but with an effect size (ε^2^) that is 0 (Supplementary Fig. 3). After correcting for multiple tests, no statistically significant differences were identified between pairwise datasets (Fig. 4a). Overall, these results indicate that no appreciable difference in GC content exist in regions with retained heterozygosity. This pattern could be due to the small number of genomic regions with retained heterozygosity examined (N = 15), to subtle differences in GC content along chromosome arms in this species, or to the reduced GC content and apparent AT-biased mutation patterns in snake genomes ^[13]^. Future investigations that sample more genome-wide loci or that leverage a higher quality reference genome for king cobra will be better positioned to localize regions of retained heterozygosity and confirm their location in telomeric regions.

## References

1. Galtier, N., Piganeau, G., Mouchiroud, D. & Duret, L. GC-Content Evolution in Mammalian Genomes: The Biased Gene Conversion Hypothesis. *Genetics* **159**, 907–911 (2001).

2. Kong, A. *et al.* A high-resolution recombination map of the human genome. *Nat. Genet.* **31**, 241–247 (2002).

3. Jensen-Seaman, M. I. *et al.* Comparative Recombination Rates in the Rat, Mouse, and Human Genomes. *Genome Res.* **14**, 528–538 (2004).

4. Meunier, J. & Duret, L. Recombination Drives the Evolution of GC-Content in the Human Genome. *Mol. Biol. Evol.* **21**, 984–990 (2004).

5. Myers, S., Bottolo, L., Freeman, C., McVean, G. & Donnelly, P. A Fine-Scale Map of Recombination Rates and Hotspots Across the Human Genome. *Science* **310**, 321–324 (2005).

6. Schield, D. R. *et al.* The origins and evolution of chromosomes, dosage compensation, and mechanisms underlying venom regulation in snakes. *Genome Res.* **29**, 590–601 (2019).

7. Schield, D. R. *et al.* Snake Recombination Landscapes Are Concentrated in Functional Regions despite PRDM9. *Mol. Biol. Evol.* **37**, 1272–1294 (2020).

8. Suryamohan, K. *et al.* The Indian cobra reference genome and transcriptome enables comprehensive identification of venom toxins. *Nat. Genet.* **52**, 106–117 (2020).

9. Kruskal, W. H. & Wallis, W. A. Use of Ranks in One-Criterion Variance Analysis. *J. Am. Stat. Assoc.* **47**, 583–621 (1952).

10. Critchlow, D. E. & Fligner, M. A. On distribution-free multiple comparisons in the one-way analysis of variance. *Commun. Stat. - Theory Methods* **20**, 127–139 (1991).

11. Benjamini, Y. & Hochberg, Y. Controlling the False Discovery Rate: A Practical and Powerful Approach to Multiple Testing. *J. R. Stat. Soc. Ser. B Methodol.* **57**, 289–300 (1995).

12. Kelley, T. L. An Unbiased Correlation Ratio Measure. *Proc. Natl. Acad. Sci.* **21**, 554–559 (1935).

13. Castoe, T. A. *et al.* The Burmese python genome reveals the molecular basis for extreme adaptation in snakes. *Proc. Natl. Acad. Sci.* **110**, 20645–20650 (2013).

## Supplementary Figures

**Supplementary Figure 1. (a)** Parthenogenetically produced *Ophiophagus hannah* offspring exhibiting developmental deformities. **(b)** Parthenogenetically produced *O. hannah* offspring exhibiting cranial deformity. Photographs courtesy of Romilly van den Bergh.

**Supplementary Figure 2.** An alluvial plot showing fate of maternal heterozygous loci (N = 278) in both offspring. 0/0, 0/1, and 1/1 encode homozygous and heterozygous loci based on the reference genome (0) and alternative alleles (1). Please note that this figure does not trace the combinatorial fates of different alleles in the two offspring, but rather provides an overview of the proportions of loci in each offspring.

**Supplementary Figure 3.** Distributions of GC content in the regions surrounding loci with retained heterozygosity in both offspring, regions surrounding loci with lost heterozygosity in both offspring, and randomly permutated datasets of regions surrounding loci homozygous in all samples.
